# PROTOCOL: Effects of neonatal nutrition interventions on neonatal mortality and child health and development outcomes: A systematic review

**DOI:** 10.1002/cl2.1021

**Published:** 2019-07-24

**Authors:** Aamer Imdad, Deepika Ranjit, Gamael Saint Surin, Sarah Lawler, Abigail A. Smith, Zulfiqar A. Bhutta

**Affiliations:** ^1^ Department of Pediatric SUNY Upstate Medical University Syracuse New York; ^2^ Department of Public Health and Preventive Medicine SUNY Upstate Medical University Syracuse New York; ^3^ Health Science Library SUNY Upstate Medical University Syracuse New York; ^4^ Department of Pediatrics Aga Khan University Karachi Pakistan; ^5^ Department of Global Health Hospital for Sickkids Toronto Canada

## 1 BACKGROUND

### 1.1 The problem, condition or issue

The decline in rates of neonatal (age 0–28 days) mortality has been slower than the decline in child mortality between 1990 and 2016 (Alkema, Chao, You, Pedersen, & Sawyer, 2014; Bhutta et al., 2015). Neonatal mortality accounted for 46% of child mortality in 2016 compared to 40% of all under‐five mortality rates in 1990 (WHO, 2017a). Globally, the percentage of neonatal mortality is the highest in South Asia and Sub Saharan Africa (Alkema et al., 2014). Optimal nutritional support during neonatal period is vital to the short and long term survival of the newborn (Bhutta et al., 2013; WHO, 2017b). Poor nutritional status of neonates is a major cause of illness and can lead to poor growth, increased risk of infection, bleeding, and neonatal death (Bhutta et al., 2013; WHO, 2017b). The risk of morbidity and mortality during neonatal period is higher in low and middle‐income countries (LMICs) where many birth happen at home and the prevalence of maternal malnutrition and incidence of low birth weight (birth weight less than 2500 g) and preterm birth (gestational age <37 weeks) is high (Bhutta et al., 2013; Lee et al., 2017; WHO, 2017b). This review will focus on selective nutritional interventions during neonatal periods in LMICs.

The approach to nutritional management of newborn depends on maternal nutritional status, co‐morbidities during pregnancy (such as gestational diabetes), pregnancy duration (term vs. preterm birth), events at birth (such as birth asyphaxia), birth weight (low birth weight vs. normal birth weight), and available resources for postpartum care of the mother and the baby (such as skill birth attendant, home vs. facility birth, availability of neonatal intensive care, etc.; Bhutta et al., 2013; WHO, 2015; WHO 2017a; WHO, 2017b). The most important nutritional intervention at birth is breastfeeding and this will be covered in a separate Campbell review of this series. There are number of other nutritional interventions that have been proposed in addition to breastfeeding and it is beyond the scope of this review to comprehensively evaluate all the possible nutritional interventions during the neonatal period. We plan to review the following three interventions: neonatal vitamin A supplementation, oral dextrose gel supplementation, and probiotic supplementation during neonatal period in LMICs. Below in this section and rest of the introduction, we describe the rationale for choosing these interventions and why it is important to do this review.

#### 1.1.1 Neonatal vitamin A deficiency

Globally, about 190 million children and 19.1 pregnant women are vitamin A deficient based on serum retinol levels (i.e., serum retinol less than 0.70 μmol/L; WHO, 2009a). Vitamin A deficiency (VAD) is most prevalent in South Asia and Africa (Stevens et al., 2015). VAD is associated with increased risk of blindness, infections, and mortality (Imdad, Mayo‐Wilson, Herzer, & Bhutta, 2017). Most of the newborns are vitamin A deficient and rely on supplementation from maternal breast milk (Haider, Sharma, & Bhutta, 2017). High prevalence of maternal VAD in LMICs increases the risk of neonatal VAD. There has been interest in vitamin A supplementation during neonatal period to assess if it reduces risk of illness and death (Haider et al., 2017; WHO, 2009b) as it has been shown to reduce morbidity and mortality in children 6–59 months of age (Imdad et al., 2017).

#### 1.1.2 Hypoglycemia during the neonatal period

Hypoglycemia is common during immediate neonatal period (Kaiser et al., 2015). Recurrent, severe, and persistent hypoglycaemia might lead to brain damage (Kaiser et al., 2015; McKinlay et al., 2017; Thornton et al. 2015). About 10–15% of otherwise healthy newborns have low blood sugars and the rate is much higher among infants with additional risk factors such as: large for gestational age, small for gestational age, low birth weight, preterm birth, infant of diabetic mother, and newborns with perinatal asyphaxia (Thompson‐Branch & Havranek, 2017). Additional risk factors for neonatal hypoglycaemia include neonatal sepsis, prolonged labor, and maternal medication use such as beta agonists and beta blockers (Thompson‐Branch & Havranek, 2017). The definition of hypoglycaemia is controversial and there is limited evidence to show that blood sugars below a certain level leads to long term brain damage. The American Academy of Pediatrics consider hypoglycaemia as blood sugar below 47 mg/dL (2.61 mmol/L); however, other societies such as Pediatric Endocrine Society consider hypoglycemia as blood sugars levels less than 50 mg/dL (2.77 mmol/L; Thompson‐Branch & Havranek, 2017; Thornton et al., 2015). The initial recommended intervention to treat early neonatal hypoglycaemia is to offer feeding in the form of breastfeeding followed by formula feeding if breastfeeding is unsuccessful. Persistent hypoglycaemia may require IV dextrose supplementation and admission to neonatal intensive care unit (Thompson‐Branch & Havranek, 2017; Thornton et al., 2015). In LMICs, where a significant proportion of births happen at home and incidence of low birth weight and preterm birth is high, prevention and treatment of hypoglycaemia might be challenging (Singhal et al., 1991; Singhal, Singh, & Paul, 1992; WHO, 2017b; Williams, 1997). The instruments to test blood sugars might not be available in low resource settings and in case blood testing is available, IV dextrose and facility of intensive care unit might not be available to treat persistent and severe hypoglycemia. Recent studies have tested simple interventions such as oral dextrose gel to prevent hypoglycaemia in his risk newborns and treat known hypoglycaemia (Hegarty et al., 2016; Weston et al., 2016).

#### 1.1.3 Neonatal sepsis and necrotizing enterocolitis

Neonatal sepsis and necrotizing enterocolitis (NEC) are neonatal morbidities that can be fatal (Oza, Lawn, Hogan, Mathers, & Cousens, 2015; WHO, 2017b). Neonatal sepsis is the presence of an infectious agent leading to systemic illness. Bacterial sepsis is common in LMICs and is a significant risk factors of morbidity and mortality (WHO, 2017aa). NEC is a condition that occurs in newborns and can lead to injury to bowel. The extent of injury may vary from mucosal injury to full thickness bowel wall injury. It happens most commonly in preterm babies especially extremely preterm babies (AlFaleh & Anabrees, 2014; Patel & Denning, 2015). Multiple factors lead to development of NEC in preterm infants including altered bacterial gut flora affecting the protective intestinal barrier, decreased intestinal motility and the increased susceptibility for inflammation and infections in preterm infants (Patel & Denning, 2015). Recent studies have shown that imbalance between commensal bacteria and pathogenic bacteria (dysbiosis) makes the babies vulnerable to pathogenic bacterial growth in the intestine causing inflammation that might lead to neonatal sepsis and/or NEC (Arrieta, Stiemsma, Amenyogbe, Brown, & Finlay, 2014; Deshmukh et al., 2014; Gewolb, Schwalbe, Taciak, Harrison & Panigrahi, 1999; Panigrahi et al., 2017). There is an increasing interest in correction of dysbiosis by probiotics to prevent NEC and neonatal sepsis and data from early studies from developed countries is encouraging (AlFaleh & Anabrees, 2014; Panigrahi et al., 2017).

### 1.2 The intervention

#### 1.2.1 Neonatal vitamin A supplementation

Vitamin A is a term used for a subclass of the family of fat soluble compounds: retinoic acids. It is found in nature in two forms: provitamin A carotenoids and preformed vitamin A essential. Plant based foods are the source of provitamin A carotenoids (Beta‐carotene is the most commonly known) and animal based foods are the sources of preformed vitamin A (Bates, 1995; Haider & Bhutta, 2011). Plant based foods may not be an adequate source of vitamin A as the gastrointestinal conversion ratio from carotenoid‐to‐retinol varies from 6:1 to 26:1. VAD may therefore exist in areas even when there is high consumption of plant based foods such as in South Asia and Africa (Imdad et al., 2017; Stevens et al., 2015). Vitamin A from animal sources (retinol, retinal, retinoic acid, and retinyl esters) is the most active form and synthetic vitamin A retinol has been used in most of intervention trials in the past (Haider & Bhutta, 2011; Imdad et al., 2017).

#### 1.2.2 Oral dextrose gel supplementation during neonatal period

Dextrose gel is a thickened aqueous solution that contains concentrated simple carbohydrate. It can be administered by direct application to oral, buccal, or sublingual mucosa and can increase blood sugars rapidly by absorption from highly vascularized and thin mucus membranes of oral mucosa (Hegarty et al., 2016). Dextrose gel is a low cost, non‐proprietary intervention and can be prepared in hospital pharmacies. The typical ingredients include water, glucose, a gelling agent, and preservatives (Hegarty et al., 2016). The decision to use dextrose gel in a neonate should be taken on individual basis and should be avoided in neonates with compromised mental status (Hegarty et al., 2016; Weston et al., 2016).

#### 1.2.3 Probiotic supplementation during neonatal period

Prebiotics are supplements that promote the growth of commensal bacteria (AlFaleh & Anabrees, 2014; Panigrahi et al., 2017). Probiotics contain live bacteria that enrich pool of commensal bacteria (AlFaleh & Anabrees, 2014; Millar, Wilks, & Costeloe, 2003; Panigrahi et al., 2017). Synbiotics are a combination of prebiotics and probiotics and might have synergistic effect (Johnson‐Henry, Abrahamsson, Wu, & Sherman, 2016; Panigrahi et al., 2017). These supplements are meant to optimize gut health and their hypothesized mechanisms of actions include enhanced gut barrier function, inhibition of gut colonization with pathogenic bacteria, improvement in colonization with healthy commensals bacteria that protect the infant from enteropathogenic infection through production of acetate, enhance innate immunity, and increase maturation of the enteric nervous system (Rao, Athalye‐Jape, Deshpande, Simmer, & Patole, 2016). Recent data have shown that probiotic supplements can prevent incidence of NEC in preterm babies (AlFaleh & Anabrees, 2014; Millar et al., 2003; Patel & Denning, 2015; van den Akker, van Goudoever, Szajewska, Embleton, & Hojsak, 2018). There are also data on use of probiotocs/synbiotics for prevention of neonatal sepsis (Panigrahi et al., 2017; Rao et al., 2016). The most commonly used strains in probiotics include *Lactobacillus* and *Bifidobacterium* (Rao et al., 2016).

### 1.3 How the intervention might work

#### 1.3.1 Neonatal vitamin A supplementation

Vitamin A has an effect on cell differentiation and helps maintain normal functioning of epithelial cells (Bates, 1995; Bhutta et al., 2013; Haider & Bhutta, 2011). It is considered anti‐infective because it helps to maintain the protective epithelial barrier of the skin and mucosa which protects the body from infections. Vitamin A helps in the regeneration of the epithelium therefore maintaining the integrity of the body's first line of defense preventing infections in newborns (McCullough, Northrop‐Clewes, & Thurnham, 1999; Wolbach, 1933). Synthetic vitamin A supplementation has been shown to reduce morbidity and mortality in children 6 to 59 months of age (Imdad et al., 2017). The potential side effects of synthetic vitamin A supplementation include vomiting and bulging fontanelle (Haider & Bhutta, 2011; Haider et al., 2017; Imdad et al., 2017; Imdad, Ahmed, & Bhutta, 2016). Excess vitamin A supplementation can cause toxicity that presents in the form of a bulging fontanelle in children under 1 year, headaches, vomiting, diarrhea, loss of appetite, and irritability (Haider et al., 2017; Imdad et al., 2017).

#### 1.3.2 Oral dextrose gel supplementation during neonatal period

The absorption of dextrose gel from oral mucosa leads to entry of glucose into lingual veins and to the internal jugular vein avoiding the first past effect of liver from the portal circulation. Such absorption provides almost immediate delivery of glucose to systematic circulation. If proven effective in preventing and treating hypoglycemia, dextrose gel can avoid the need of intravenous glucose and separation of baby from mother (Hegarty et al., 2016; Weston et al., 2016). The intervention is simple enough that it does not require special skills (such as IV placement) and can be administered by community, lay health workers, and mother themselves. The potential adverse effects include vomiting, choking, gagging, respiratory distress, and delay of treatment for severe hypoglycaemia (Hegarty et al., 2016; Weston et al., 2016).

#### 1.3.3 Probiotics supplementation during neonatal period

Newborns and preterm babies have immature intestines free of normal commensal bacteria and are more likely to develop NEC and sepsis due to growth of pathogenic bacteria in the intestines (AlFaleh & Anabrees, 2014; Patel & Denning, 2015; Rao et al., 2016). Probiotics are used to proactively colonize the intestines with bacteria like lactobacillus which are known to be beneficial (Millar et al., 2003; Patel & Denning, 2015). Probiotics therefore reduce the growth of pathogenic bacteria which leads to NEC and sepsis. It also increases gut immunity by increasing IgA levels with the help of normal flora which help maintain the mucosal barrier as well (Patel & Denning, 2015). These protective mechanisms also reduce intestinal permeability producing a protective mucosal barrier against bacteria and increase the production of anti‐inflammatory cytokines (Deshpande, Jape, Rao, & Patole, 2017; Millar et al., 2003). Probiotics are especially protective in preterm babies with immature guts and neonates on antibiotics which affects the normal flora of the intestines allowing for colonization by pathogenic bacteria causing NEC. Prebiotics and probiotics can be given together in the form of a synbiotic to improve the gut flora and it can potentially reduce all‐cause neonatal mortality (Johnson‐Henry et al., 2016; Panigrahi et al., 2017). Probiotics are considered safe; however, there are concerns regarding probiotic supplementation in extremely premature, immunocompromised neonates and few cases of neonatal sepsis have been reported that were thought to be caused by probiotics (Dani et al., 2016).

### 1.4 Why it is important to do the review

#### 1.4.1 Neonatal vitamin A supplementation

The randomized trials on neonatal vitamin A supplementation have produced conflicting results with some studies (mostly from South Asia) showing a mortality benefit while no major benefit in other studies (mostly from Africa) (Haider et al., 2017) and some studies showing even an increased risk of infant mortality in certain population (Smith et al., 2016). The exact reason for this difference in results is not clear and previous reviews (Gogia & Sachdev, 2009; Haider et al., 2017) and a WHO technical consultation (WHO, 2009a) have hypothesized that certain factors might explain the difference in results from different studies. The hypothesized factors include timing of supplementation (early supplementation within 96 hr vs. late supplementation), age at death (1 month vs. 6 months vs. 12 months), infant vitamin A status (vitamin A deficient vs. vitamin A sufficient), maternal vitamin A status (vitamin A deficient vs. vitamin A sufficient), infant vaccine history (vaccinated vs. unvaccinated), sex of newborn, timing of initiation of breastfeeding, duration of exclusive breastfeeding, timing of introduction of complementary feedings, and season when supplemented (e.g., high/low disease transmission, birth weight [very low birth weight, low birth weight, and normal]). We plan to attempt the subgroup analyses that have not been done in the previous reviews (Gogia & Sachdev, 2009; Haider et al., 2017) and also attempt meta‐regression analysis to assess if certain factors explain the heterogeneity in the published studies. We also plan to include studies on low birth weight and preterm infants as the Cochrane review focused on mostly term infants (Haider et al., 2017). We also plan to look at long term neurodevelopmental outcomes of supplementation during the neonatal period as recent data on long term outcomes are available from trials done earlier (Ali et al., 2017).

#### 1.4.2 Oral dextrose gel supplementation during neonatal period

Oral dextrose as a treatment of hypoglycemia and prevention of hypoglycemia in high risk neonates have been evaluated in two cochrane reviews (Hegarty, Harding, Crowther, Brown, & Alsweiler, 2017; Weston et al., 2016). The review by Weston et al. (2016) addressed treatment of neonatal hypoglycemia and included two studies, one from New Zealand and other from Ireland and did not show any major difference in episodes of hypoglycemia episodes between the two study groups. The review by Hegarty et al. (2016) addressed prevention of hypoglycemia in high risk neonates and included one study from New Zealand. The included study showed significant reduction in hypoglyemia in the intervention compared to control [Risk ratio 0.76; 95% confidence interval 0.62–0.94). No randomized study was available from LMICs in either the two reviews mentioned above. Our objective is to consider both randomized and nonrandomized observational studies with a control arm. We also anticipate that use of dextrose might be more beneficial in LMICs as the incidence of neonatal hypoglycemia might be higher because of higher rates of preterm birth and low birth weights births.

#### 1.4.3 Probiotics/prebiotics/synbiotics supplementation during neonatal period

The effect of probiotic supplementation for prevention of NEC and neonatal sepsis have been assessed in previous reviews (AlFaleh & Anabrees, 2014; Rao et al., 2016; van den Akker et al., 2018). Most of the included studies in these reviews were conducted in developed countries in facility based settings. A recent community based study conducted in India showed that use of synbiotics (probiotic+prebiotics) prevents neonatal sepsis/mortality (Panigrahi et al., 2017). This trial however included neonates with gestational age >35 weeks and birth weight >2000 g. The risk of sepsis might be higher in very preterm and very low birth weight babies; however, these babies might not survive in community settings without advanced care such as provided in a neonatal intensive care unit. Our objective is to include randomized and nonrandomized studies from LMICs to assess the effect of probiotic supplementation on prevention of neonatal morbidity and mortality.

## 2 OBJECTIVES

Primary objectives

To determine the efficacy and effectiveness of the following interventions on neonatal morbidity and mortality.

1. Oral vitamin A supplementation
2. Oral dextrose gel supplementation
3. Probiotic supplementation

The term efficacy refers to how an intervention works under the ideal conditions and effectiveness refers to how an intervention works under real world conditions.

## 3 METHODOLOGY

### 3.1 Criteria for including and excluding studies

#### 3.1.1 Types of study designs

We will consider experimental or quasi‐experimental studies to determine the efficacy and effectiveness of included interventions.

We will consider the following study designs.

● Randomized controlled trials (RCTs), where participants were randomly assigned, individually or in clusters, to intervention and comparison groups. Cross‐over designs will be eligible for inclusion.
● Quasi‐experimental designs, which include
  ○ Natural experiments: studies where non‐random assignment is determined by factors that are out of the control of the investigator. One common type includes allocation based on exogenous geographical variation.
  ○ Controlled before‐after studies (CBA), in which measures were taken of an experimental group and a comparable control group both before and after the intervention. We also require that appropriate methods were used to control for confounding, such as statistical matching (e.g., propensity score matching, or covariate matching) or regression adjustment (e.g., difference‐in‐differences, instrumental variables).
  ○ Regression discontinuity designs; here, allocation to intervention/control is based upon a cut‐off score.
  ○ Interrupted time series (ITS) studies, in which outcomes were measured in the intervention group at least three time points before the intervention and after the intervention.

The inclusion of randomized and nonrandomised studies will broaden the scope of this reviews as some of the intervention such as probiotics and dextrose supplementation are relatively new and large number of randomized studies might not be available from low and middle‐income countries. This however brings the challenge of drawing conclusion from these data as nonrandomized studies are at higher risk of selection and performance bias. We will analyze the randomize and nonrandomize separately to avoid mixing of data from these two type of studies.

#### 3.1.2 Types of participants

Participants for this study will include neonates (aged 0–28 days) from LMICs. We will include neonates regardless of their health status: this includes low birth weight and preterm babies. However, studies that focused on neonates with congenital anomalies will be excluded. We will consider studies that include older age population groups in addition to neonates provided we can disaggregate relevant data for neonatal population. For example, a study might include infants up to 6 months of age. We will include this study if the disaggregated data are available for neonates (0–28 days). Even though we plan to assess the childhood outcomes, we do not plan to include studies that recruited participants after the neonatal period.

#### 3.1.3 Types of interventions

The following interventions will be included in the review

1. Neonatal vitamin A supplementation compared to no supplementation: we will consider only oral synthetic vitamin A supplementation. There will be no restriction on the dosage and frequency of the medicine. The comparison group may include placebo or standard of care.
2. Oral dextrose gel supplementation during neonatal period compared to no supplementation: we will place no limits on dose or frequency of the dextrose supplementation. We will only include dextrose gel as intervention and exclude dextrose given in other form such as intravenous, nasogatric tube or mixed with infant formula. The comparison group may include placebo or standard of care.
3. Neonatal oral probiotics compared to no probiotic supplementation: probiotics are live microbial supplementation that are given to promote the growth of commensal gut bacteria and prevent the growth of pathogenic bacteria. Prebiotics are dietary supplements that promote the growth of commensal bacteria. Synbiotics are combination of prebiotics and probiotics (Millar et al., 2003; Patel & Denning, 2015). We will place no limits on the dose or frequency of probiotics. We will only include studies that used probiotics and synbiotics supplementation and exclude studies that used only prebiotics. Comparison groups may include placebo or standard of care.

Each of the above intervention will be summarized separately and will not be compared to each other directly or indirectly.

#### 3.1.4 Types of outcome measures

##### 3.1.4.1 Primary outcomes

The primary outcomes to be measured are

1. Neonatal mortality (death between 0 and 28 days of life)
2. All cause infant mortality at 6 months (death between 0 days to 6 months of life)
3. All‐cause infant mortality at 12 months (death between 0 days to 12 months life).

It is possible that studies may not report the outcomes in the follow up period mentioned above for the primary outcomes. If a study does not report mortality outcomes at day 28, 6 months, or 12 months, we will contact authors for data for the same. If segregated data are not available from authors, we will include mortality data as follows: mortality in first 6 weeks of life will be included as neonatal mortality at day 28; between 3 and 6 months will be included as 6 months and between 9 and 12 months will be included as 12 months.

##### 3.1.4.2 Secondary outcomes

The secondary outcomes to be measured will include

1. Sepsis specific mortality measured between 0 and 28 days, 0 days to 6 months and 0 days to 12 months of life
2. Neonatal sepsis (as defined by authors) in first 6 weeks of life
3. Necrotizing enterocolitis as defined by authors
4. Biochemical levels of micronutrients
  a. Retinol levels for vitamin A
5. Prevention of Hypoglycemia (as defined by authors) during the neonatal period
6. Treatment of Hypoglycemia (recurrence of hypoglycemia after the episode treated)
7. Any adverse reactions during the intervention period
8. Serious adverse events
9. Neurodevelopmental outcomes at 12 and 24 months and at the longest follow up.

The term neurodevelopment is a composite term that refers to cognitive, neurologic, and/or sensory outcomes. This may include intellectual disability as measured on Mental Developmental Index of the Bayley Scales of Infant Development; gross motor delay measured on Gross Motor Function Classification System; and hearing and vision loss requiring amplification devices.

In order to be eligible for inclusion in the review, a study should report at least one of the primary or secondary outcome. We will not consider this as an exclusion criterion at the screening stages but at the full text review stage.

#### 3.1.5 Duration of follow‐up

We will include all participants in eligible studies that had outcomes of interest measured. There will be no restrictions based on duration of exposure, duration of follow‐up, or timing of outcome measurement. If the duration of treatment exceeds the neonatal period (i.e., 28 days), we will consider another 2 weeks maximum but will not include studies in which the treatment goes beyond 6 weeks of supplementation. We will measure primary outcome at 28 days, 6 months, and 12 months of life. For secondary outcomes on neurodevelopment, we will consider the outcome at 12 and 24 months and at the longest follows up.

#### 3.1.6 Types of settings

We will include studies conducted in LMICs. These countries are defined as those with a gross national income (GNI) per capita of USD 1,005 or less in 2016 and lower middle‐income economies are those with a GNI per capita between USD 1,006 and 3,955 in 2016 (World Bank, 2017). We might consider studies from upper income countries if no studies are available from LMICs for an intervention.

### 3.2 Search strategy

The identification of studies will include various methods both electronic and other sources and will not be dependent on outcome of the interventions. Our PICO table (Table 1) will be used for formulating the search strategy.

**Table 1:** PICO table used for formulating the search strategy

Elements	Description
P	Neonates (aged 0–28 days), regardless of health status including low birth weight and preterm babies, from LMICs No vertical lines, only horizontal.
I	1. Oral vitamin A supplementation during neonatal period 2. Oral dextrose gel supplementation during neonatal period 3. Probiotic supplementation during neonatal period
C	Interventions will be compared to placebo, no intervention or the standard care
O	**Primary outcomes**
	• Neonatal Mortality (Death between 0 and 28 days of life)
	• All‐cause infant mortality at 6 months (Death between 0 days to 6 months of life)
	• All‐cause infant mortality at 12 months (Death between 0 days to 12 months of life)
	**Secondary outcomes**
	• Cause specific mortality
	• Neonatal sepsis
	• Necrotizing Enterocolitis (NEC)
	• Retinol levels for vitamin A
	• Hypoglycemia
	• Hypothermia
	• Any adverse reactions during the intervention period
	• Long term and Developmental outcomes

#### 3.2.1 Electronic searches

The electronic search for relevant studies will be done in databases like PubMed, EMBASE, the Cochrane Library, Cochrane Central Register for Controlled trials, Web of Science, CINHAL, Scopus, LILACS, Popline, and WHO Global Health Library.

Appendix 1 gives the search strategy for PubMed, CINHAL, LILACS, SCOPUS, and CENTRAL that include key words and MeSH terms as appropriate. This includes search strategy for Population (neonates) and Interventions. We plan to run searches for each intervention separately. We will first run the search for population, which is the same for each intervention. Then we will run the search for each individual intervention. We will combine both searches by using “AND” and keep the searches in a separate EndNote file.

An example of a search strategy for vitamin A is as follows:

((((("Vitamin A"[Mesh]) OR (Vitamin A[tiab] OR Aquasol A[tiab] OR Retinol[tiab] OR All Trans Retinol[tiab] OR All‐Trans‐Retinol[tiab] OR Vitamin A1[tiab] OR Vitamin A 1[tiab] OR 11‐cis‐Retinol[tiab] OR 11 cis Retinol[tiab] OR Tretinoin[tiab]) AND Supplement*[tiab])) AND (("Infant"[Mesh] OR "Premature Birth"[Mesh]) OR (Neonat*[tiab] OR neo nat*[tiab]) OR (newborn* OR new Born*[tiab] OR newly born*[tiab]) OR (preterm[tiab] OR preterms[tiab] OR pre term[tiab] OR pre terms[tiab]) OR (premature*[tiab] AND (birth*[tiab] OR born[tiab] OR deliver*[tiab])) OR (low[tiab] AND (birthweight*[tiab] OR birth weight*[tiab])) OR (lbw[tiab] OR vlbw[tiab] OR elbw[tiab]) OR infant*[tiab] OR (baby[tiab] OR babies[tiab])))) NOT ("Animals"[Mesh] NOT ("Animals"[Mesh] AND "Humans"[Mesh]))

We do not plan to apply any restrictions on searches based on outcomes, study design or language. There will be no restrictions on date of publication. We will restrict studies to Human studies only.

#### 3.2.2 Searching other resources

Other resources will include search for ongoing trials at “www.clinicaltrials.gov” and WHO's ICTRP trials database. We will also search websites of international agencies such as WHO, (including WHO's Reproductive Health Library) UNICEF, Global Alliance for Improved Nutrition, International Food Policy Research Institute, International Initiative for Impact Evaluation (3ie), Nutrition International, UNICEF, World Bank, USAID, and affiliates (e.g., FANTA, SPRING) and the World Food Programme.

Gray literature searches will include Nutrition International (NI), Global Alliance for Improved Nutrition (GAIN), International Food Policy and Research Institute (IFPRI), and WHO library database (WHOLIS).

We will search the reference lists of all included studies. We will do citation searches of included studies in Google Scholar and Web of Science. We also will search the reference sections of previously published systematic reviews and the latest published studies. We will contact the experts and authors of the newest published studies to ask about any additional studies.

### 3.3 Description of methods used in primary research

We expect that majority of the included studies will be randomized or cluster randomized. For example, a study published by Katz et al. (Klemm et al., 2008) was a cluster randomized trial of neonatal and maternal vitamin A supplementation or placebo. As mothers also received vitamin A supplementation in this trial, we will include the data on neonatal vitamin A supplementation in a way that there is no contamination of intervention from maternal supplementation. This means that we will include the data as follows: maternal vitamin A+neonatal vitamin A vs. maternal placebo+neonatal placebo.

### 3.4 Criteria for determination of independent findings

It is expected that authors might report results of a study in multiple publications. We will code such trials as a single study to avoid double counting of the data and include all the relevant outcomes decided a priori for this review. If a pilot study was done before the larger study, we will include the two studies separately unless the data from pilot study was included in the main trial. When a clinical trial registration number is available for a study, we will search that number on PubMed to locate all the published studies linked to that trial number.

### 3.5 Details of study coding categories

The data from included studies will be abstracted into a standardized data abstraction form by two authors. We will extract data in duplicates and any discrepancies will be resolved by discussion first. A third reviewer (ZAB) will be consulted if the conflict exists after initial discussion.

The data extraction sheet will have the following information.

● General study information: authors, publication year, study design
● Study setting: World Bank region, country, World Bank income level, city/town, urban/urban slum/rural/mixed setting, duration of data collection, date of data collection
● Study population: sample size recruited, sample size analyzed, female (%), description of participants (i.e., inclusion/exclusion criteria applied to recruitment)
● Intervention characteristics: type of intervention, duration of intervention, unit of randomization (where applicable), dose, frequency of provision, duration of follow up, attrition rate
● Funding source
● Quality assessment.

Each quantitative outcome sheet will contain the following:

● Subgroup (if applicable)
● Subgroup sample size
● Outcome type
● Outcome units
● Outcomes:
  ○ Outcome measure treatment group
  ○ Outcome measure comparison group
  ○ Standard deviation
● Effect size:
  ○ Effect measure
  ○ 95% confidence interval
  ○ *P*‐value of effect measure
  ○ Standard error or t‐statistic.

To avoid reviewer bias, we plan to predetermine the preference for certain data for certain outcomes. For example, for mortality outcomes, we will give preference to denominators in the following order: number with definite outcome known, number randomized, and child‐years. For morbidity data such as neonatal sepsis where both survivors and non‐survivors may have contributed data, we will give preference to child years, number with definite outcome known, and number randomized. For randomized trials, we will give preference to data that requires the least manipulation by authors or inference by reviewers. We will extract the raw values (for example, means, and standard deviations) and build the intention to treat analysis where applicable.

We anticipate that cause‐specific morbidity or mortality data might not be readily available as febrile illness due to respiratory, urinary or central nervous system infection are categorized under a broader term of neonatal sepsis (WHO, 2017b).

#### 3.5.1 Unit of analysis issues

As we plan to include multiple interventions, all interventions and, within those interventions, outcomes will be meta‐analyzed separately. We will analyze randomized and nonrandomized studies separately.

For randomized trials, we will meta‐analyze individual and cluster randomized trials in the same analysis. We will assess analyses in the cluster randomized trials to ensure that clustering has been appropriately accounted for within the analysis of the primary study, such that study precision is not over or under‐estimated within our analysis. If authors adjust for cluster randomization, no further adjustment will be done. In case a cluster randomized study is not adjusted by primary authors, we will adjust effect estimates by using the mean cluster size (M) and the intra‐cluster correlation coefficient (ICC) to calculate the design effect as follows: design effect = 1 + (M − 1) ICC. We will then use the design effect to adjust the study data such that a trial is reduced to its effective sample size or standard error of summary estimate is inflated. We will use the ICC given in the published studies. If the ICC is not available from the published study, we will contact the authors for the same. If the ICC is not available from the authors, we will use ICC from the similar studies done in the similar region and on the similar population or will take it from the previously published reviews (Haider et al., 2017).

#### 3.5.2 Multiple‐arm trials

We will include studies that have multiple intervention arms, but we will only include the arms that are eligible for the review. We will select one pair (with appropriate intervention and control group) that satisfy the inclusion criteria of the review and exclude the rest. In case there are more than two groups eligible for inclusion, we will combine these groups into a single pair‐wise comparison. In multiple‐arm trials using two different doses of the same intervention, we will combine the two groups to avoid double counting the participants in the control group.

Any missing data will be noted including loss to follow‐up and dropouts. The reasons for the missing data will be taken from the studies and if it is not mentioned in the studies, the authors will be contacted. If authors reported the adjusted values for missing data, we will use the adjusted values.

We will assess each of the included study for risk of bias according to the Cochrane Effective Practice and Organisation of Care (EPOC) guidelines (EPOC, 2017). EPOC guidelines include the following standardized criteria for assessing bias of randomized, nonrandomized, and controlled before‐after studies (EPOC, 2017):

● Random sequence generation
● Allocation concealment
● Baseline outcome measurements similar
● Baseline characteristics similar
● Incomplete outcome data
● Knowledge of the allocated interventions adequately prevented during study
● Protection against contamination
● Selective outcome reporting
● Other risks of bias (e.g., bias in measurement: validity and reliability of the measures used).

For ITS studies, the following criteria will be considered (EPOC, 2017):

● Intervention independent of other changes
● Shape of intervention effect pre‐specified
● Intervention unlikely to affect data collection
● Knowledge of the allocated interventions adequately prevented during study
● Incomplete outcome data
● Selective outcome reporting
● Other risks of bias (e.g., bias in measurement: validity and reliability of the measures used).

We will also use the Cochrane risk of bias (ROB) tool (Higgins & Green, 2011) in addition to EPOC guidelines for randomized studies, including cluster randomized trials and step‐wedge designs. The Cochrane risk of bias include the following items

● Selection bias: random sequence generation and allocation concealment
● Performance bias: blinding of participants and personnel
● Detection bias: blinding of outcome assessment
● Attrition bias: incomplete outcome data
● Reporting bias: selective reporting
● Other sources of bias.

Two authors will independently perform the risk of bias assessments for each study. A third reviewer will resolve any disagreements. An overall score will not be provided.

### 3.6 Statistical procedures and conventions

We will use software Review Manager 5.3 and Stata to conduct the statistical analysis. For randomized trials, we will follow the intention to treat analysis (ITT). If ITT is not available and author reports the analyses as specified in the protocol, we will reconstruct the data to create an ITT analysis.

We will perform meta‐analysis for synthesis of quantitative data when the included studies have comparable participants, interventions, and outcomes. We will not assess the effect on outcome across the intervention such as done in network‐meta‐analysis. Each intervention will be analyzed separately. The outcome data from individual studies will be converted in the same format (e.g., mean difference and standard deviation for continuous data) and the direction/scale of effect adjusted in a way that an increase/decrease always indicates improvement or deterioration of an indicator. We will analyze continuous and dichotomous data separately. For dichotomous outcomes, results will be presented as summary risk ratios with 95% confidence interval (CI). We will combine risk ratios (events per child) and rate ratios (events per child year) for incidence data because of their similar interpretation and scale. For continuous outcomes, we will present the summary results as mean difference with 95% CI when data are available in the same scale across the studies. We will use the standardized mean difference with 95% CI when data are presented in different scales across the studies.

We will use random effect model to account for expected heterogeneity in the intervention, comparisons, or setting within studies included in a given synthesis. When there is substantial methodological or statistical heterogeneity among included studies, we will summarize the findings in narrative or table form and avoid the meta‐analysis. We will use generic inverse variance method of meta‐analysis for fixed effect models and random effect model. This method of meta‐analysis gives weight to studies based on their variance in a way that a study with low variance gets a high weight and vice versa.

Statistical heterogeneity will be assessed using *τ* ^2^, *I* ^2^, and significance of the *χ* ^2^ test; we will also assess heterogeneity visually using forest plots. Based on prior theory and clinical knowledge, we expect clinical and methodological heterogeneity in effect sizes in this literature. Therefore, we will attempt to explain any observed statistical heterogeneity using subgroup analysis

If studies report adjusted and unadjusted estimates, we will use the most adjusted estimates.

We will interpret the results of meta‐analysis based on *p* value (a value <.5 will be considered statistically significant) and report both significant and nonsignificant results. For subgroup analysis, we will use an interaction tests to determine if there is a relevant difference in effect across subgroups.

A funnel plot and its symmetry will be used to assess publication bias if the number of included for an intervention was more than 10. If the funnel plot is suggestive for publication bias, we will further investigate with Egger's test (Higgins & Green, 2011).

For each individual outcome, we shall assess the quality of the evidence using the GRADE approach, which involves consideration of within‐study risk of bias (methodological quality), directness of evidence, heterogeneity, precision of effect estimates and risk of publication bias (Guyatt et al., 2011). We will rate the quality of the body of evidence for each key outcome as “high”, “moderate”, “low” or “very low” Table 2. Nonrandomized studies will initially be rated as “low” quality. If there are no serious methodological flaws, we will upgrade the evidence for studies with a large magnitude of effect; presence of a dose response relationships; and effect of plausible residual confounding.

**Table 2:** Quality of evidence, as determined by the GRADE criteria

Quality	Description
Very low	Any estimate of effect is uncertain
Low	Further research is very likely to have an important impact on our confidence in the estimate of effect and is likely to change the estimate
Moderate	Further research is likely to have an important impact on our confidence in the estimate and may change the estimate
High	Further research is very unlikely to change our confidence in the estimate of effect

### 3.7 Subgroup analysis and investigation of heterogeneity

We plan to conduct subgroup analysis if data are available from at least three studies per subgroup of interest,

Subgroup analysis for neonatal Vitamin A supplementation

1. Timing of vitamin A supplementation (early supplementation within 96 hr vs. late supplementation after 96 hr)
2. Infant vitamin A status (vitamin A deficient vs. vitamin A sufficient)
3. Maternal vitamin A status (vitamin A deficient vs. vitamin A sufficient)
4. Infant vaccine history (vaccinated vs. unvaccinated)
5. Sex of newborn
6. Timing of initiation of breastfeeding (within 24–48 hr vs. after 48 hr)
7. Duration of exclusive breastfeeding (less than 3 months vs. 3–6 months vs. greater than 6 months)
8. Timing of introduction of complementary feedings (less than 4 months vs. between 4 and 6 months vs. at or after 6 months)
9. Birth weight (low birth weight, that is, birthweight less than 2,500 vs. normal birthweight, that is, birthweight between 2,500 and 4,000 g)
10. Gestational age: Full term (gestational age greater than 37 weeks) versus preterm (gestational age less than 37 weeks)
11. Season when supplemented (e.g., high/low disease transmission)
12. Geography: South Asia versus Africa versus Latin America
13. Control group neonatal mortality: Neonatal mortality equal or greater than 30/1,000 versus Neonatal Mortality less than 30/1,000.

Subgroup analysis 1–10 for vitamin A supplementation will be based on within study subgroup analysis of the data. In case, a study does not report the disaggregated data for our a priori subgroup analysis, we will write authors for the same. If authors did not attempt the mentioned analysis, we will ask for the raw data so that we can conduct the desired analysis. Subgroup analyses 12–13 will be at the study level.

The subgroup analysis mentioned above are based on hypotheses generated in WHO consultation group meeting about the efficacy of vitamin A supplementation (WHO, 2009b).

Oral dextrose gel supplementation

1. Gestational age: term and postterm versus late preterm 35 to 36 weeks versus moderately preterm 30 to 34 weeks versus extremely preterm <30 weeks.
2. Dose: Equal or less than 200 mg/kg versus greater than 200 mg/kg.
3. Frequency: one versus more than one dose.
4. Time of administration: less or equal than 1 hr of age versus after 1 hr of age versus after 2 hr of age.

The first subgroup analysis for dextrose will be based on within study subgroup analysis and rest will be at the study level.

Neonatal probiotic supplementation

1. Gestational age: term and postterm versus late preterm 35 to 36 weeks versus moderately preterm 30 to 34 weeks versus extremely preterm <30 weeks
2. Strains used in probiotics
3. Birth weight (very low birth weight vs. low birth weight vs. normal)

The above subgroup analysis will be based on within study subgroup analysis from the individual studies.

The interpretation of subgroup analyses is challenging as subgroup analyses are observational in nature. We will compare the confidence intervals between the two subgroup analyses and an overlapping confidence interval will rule out any difference between the two subgroups. We will also use *χ* ^2^ statistical tests to assess subgroup differences and a *p* value <.1 will be considered statistically significant. We will use random effect model when doing the subgroup analyses as fixed effect model are at higher risk of showing false positive difference the two subgroups. We aim to conduct most of the subgroup analysis based on within study subgroup difference. This is a demanding task as such data might not be available from the individual studies. We will request authors for original data if within study subgroup data are nor available.

### 3.8 Sensitivity analysis

1. High quality studies vs low quality studies. The quality of study will be based on risk of bias assessment
2. Random versus fixed effect models

### 3.9 Treatment of qualitative research

We do not plan to include qualitative research.

## AUTHOR CONTRIBUTIONS

Content: Z. A. B. and A. I. Systematic review methods: A. I., D. R., and G. S. Statistical analysis: A. I. Information retrieval: A. S. and S. L.

## CONFLICT OF INTERESTS

Dr. Bhutta was involved in study on neonatal vitamin A supplementation. He will not be involved in data extraction for this study to avoid the bias in quality assessment of the study.

## APPENDIX 1

### Literature Search Strategy

#### Neonatal vitamin A supplementation search strategy

##### PubMed

((((("Vitamin A"[Mesh]) OR (Vitamin A[tiab] OR Aquasol A[tiab] OR Retinol[tiab] OR All Trans Retinol[tiab] OR All‐Trans‐Retinol[tiab] OR Vitamin A1[tiab] OR Vitamin A 1[tiab] OR 11‐cis‐Retinol[tiab] OR 11 cis Retinol[tiab] OR Tretinoin[tiab]) AND Supplement*[tiab])) AND (("Infant"[Mesh] OR "Premature Birth"[Mesh]) OR (Neonat*[tiab] OR neo nat*[tiab]) OR (newborn* OR new Born*[tiab] OR newly born*[tiab]) OR (preterm[tiab] OR preterms[tiab] OR pre term[tiab] OR pre terms[tiab]) OR (premature*[tiab] AND (birth*[tiab] OR born[tiab] OR deliver*[tiab])) OR (low[tiab] AND (birthweight*[tiab] OR birth weight*[tiab])) OR (lbw[tiab] OR vlbw[tiab] OR elbw[tiab]) OR infant*[tiab] OR (baby[tiab] OR babies[tiab])))) NOT ("Animals"[Mesh] NOT ("Animals"[Mesh] AND "Humans"[Mesh]))

##### CINHAL

(MH”Vitamin A") OR TI ("Vitamin A" OR "Aquasol A" OR Retinol OR "All Trans Retinol" OR "All‐Trans‐Retinol" OR "Vitamin A1" OR "Vitamin A 1" OR "11‐cis‐Retinol" OR "11 cis Retinol" OR Tretinoin) OR AB ("Vitamin A" OR "Aquasol A" OR Retinol OR "All Trans Retinol" OR "All‐Trans‐Retinol" OR "Vitamin A1" OR "Vitamin A 1" OR "11‐cis‐Retinol" OR "11 cis retinol" OR Tretinoin)

AND

TI (Supplement*) OR AB (Supplement*) OR MH "Dietary Supplementation" OR MH "Dietary Supplements"

AND

(MH "Infant" OR MH "Infant, Premature" OR MH "Infant, Newborn") OR TI ((Neonat* OR neo nat*) OR (newborn* OR new Born* OR newly born*) OR (preterm OR preterms OR pre term OR pre terms) OR (premature* AND (birth* OR born OR deliver*)) OR (low AND (birthweight* OR birth weight*)) OR (lbw OR vlbw OR elbw) OR infant* OR (baby OR babies)) OR AB ((Neonat* OR neo nat*) OR (newborn* OR new Born* OR newly born*) OR (preterm OR preterms OR pre term OR pre terms) OR (premature* AND (birth* OR born OR deliver*)) OR (low AND (birthweight* OR birth weight*)) OR (lbw OR vlbw OR elbw) OR infant* OR (baby OR babies))

NOT

(MH "Animals" NOT (MH "Animals" AND MH "Humans"))

Limiter: Exclude MEDLINE records

##### SCOPUS

(TITLE‐ABS("Vitamin A" OR "Aquasol A" OR retinol OR "All Trans Retinol" OR "Vitamin A1" OR "11‐cis‐Retinol" OR treinoin)) AND (TITLE‐ABS(Supplement*)) AND (TITLE‐ABS ((neonat* OR "neo nat*") OR (newborn* OR "new born*" OR "newly born*") OR (preterm OR preterm OR "pre term" OR "pre terms") OR (premature) AND (birth* OR born OR deliver*) OR (low AND (birthweight* OR "birth weight*")) OR (lbw OR vlbw OR elbow) OR infant* OR (baby OR babies))) AND NOT INDEX(medline)

##### CENTRAL

1. MeSH descriptor: [infant] explode all trees
2. MeSH descriptor: [Premature Birth] explode all trees
3. (Neonat*:ti,ab OR neo nat*:ti,ab) OR (newborn*:ti,ab OR new Born*:ti,ab OR newly born*:ti,ab) OR (preterm:ti,ab OR preterms:ti,ab OR pre term:ti,ab OR pre terms:ti,ab) OR (premature*:ti,ab AND (birth*:ti,ab OR born:ti,ab OR deliver*:ti,ab)) OR (low:ti,ab AND (birthweight*:ti,ab OR birth weight*:ti,ab)) OR (lbw:ti,ab OR vlbw:ti,ab OR elbw:ti,ab) OR infant*:ti,ab OR (baby:ti,ab OR babies:ti,ab)
4. #1 OR #2 OR #3
5. MeSH descriptor: [Animals] explode all trees
6. MeSH descriptor: [Humans] explode all trees
7. (#5 NOT (#5 AND #6))
8. supplement*:ti,ab
9. MeSH descriptor: [Vitamin A] explode all trees
10. "Vitamin A":ti,ab OR "Aquasol A":ti,ab OR Retinol:ti,ab OR "All Trans Retinol":ti,ab OR "All‐Trans‐Retinol":ti,ab OR "Vitamin A1":ti,ab OR "Vitamin A 1":ti,ab OR "11 cis Retinol":ti,ab OR "11‐cis‐Retinol":ti,ab OR Tretinoin:ti,ab
11. #9 OR #10
12. #11 AND #8
13. #12 AND #4 NOT #7
14. "accession number" near pubmed
15. #13 NOT #14

#### LILACS

(tw:(("Vitamin A"))) OR (ti:(("Aquasol A" OR retinol OR "All Trans Retinol" OR "Vitamin A1" OR "11‐cis‐Retinol" OR tretinoin))) OR (ab:(("Aquasol A" OR retinol OR "All Trans Retinol" OR "Vitamin A1" OR "11‐cis‐Retinol" OR tretinoin))) AND (ti:(supplement)) OR (ab:(supplement)) AND (tw:(Infant)) OR (tw:("Premature Birth")) OR (ti:(((neonat* OR "neo nat*") OR (newborn* OR "new born*" OR "newly born*") OR (preterm OR preterms OR "pre term" OR "pre terms") OR (premature) AND (born OR deliver*) OR (low AND (birthweight* OR "birth weight*")) OR (lbw OR vlbw OR elbw) OR (baby OR babies)))) OR (ab:(((neonat* OR "neo nat*") OR (newborn* OR "new born*" OR "newly born*") OR (preterm OR preterms OR "pre term" OR "pre terms") OR (premature) AND (born OR deliver*) OR (low AND (birthweight* OR "birth weight*")) OR (lbw OR vlbw OR elbw) OR (baby OR babies)))) AND db:("LILACS")

### Probiotics Search Strategy

#### PubMed

((((("Probiotics"[Mesh] OR "Prebiotics"[Mesh] OR "Synbiotics"[Mesh]) OR (Probiotic*[tiab] OR prebiotic*[tiab] OR synbiotic*[tiab]))) AND (("Infant"[Mesh] OR "Premature Birth"[Mesh]) OR (Neonat*[tiab] OR neo nat*[tiab]) OR (newborn* OR new Born*[tiab] OR newly born*[tiab]) OR (preterm[tiab] OR preterms[tiab] OR pre term[tiab] OR pre terms[tiab]) OR (premature*[tiab] AND (birth*[tiab] OR born[tiab] OR deliver*[tiab])) OR (low[tiab] AND (birthweight*[tiab] OR birth weight*[tiab])) OR (lbw[tiab] OR vlbw[tiab] OR elbw[tiab]) OR infant*[tiab] OR (baby[tiab] OR babies[tiab])))) NOT ("Animals"[Mesh] NOT ("Animals"[Mesh] AND "Humans"[Mesh]))

#### CINAHL Strategies

(MH "Probiotics") OR (MH "Prebiotics") OR TI (probiotic* OR prebiotic* OR synbiotic*) OR AB (probiotic* OR prebiotic* OR synbiotic*)

AND

(MH "Infant" OR MH "Infant, Premature" OR MH "Infant, Newborn") OR TI ((Neonat* OR neo nat*) OR (newborn* OR new Born* OR newly born*) OR (preterm OR preterms OR pre term OR pre terms) OR (premature* AND (birth* OR born OR deliver*)) OR (low AND (birthweight* OR birth weight*)) OR (lbw OR vlbw OR elbw) OR infant* OR (baby OR babies)) OR AB ((Neonat* OR neo nat*) OR (newborn* OR new Born* OR newly born*) OR (preterm OR preterms OR pre term OR pre terms) OR (premature* AND (birth* OR born OR deliver*)) OR (low AND (birthweight* OR birth weight*)) OR (lbw OR vlbw OR elbw) OR infant* OR (baby OR babies))

NOT

(MH "Animals" NOT (MH "Animals" AND MH "Humans"))

Limiter: Exclude MEDLINE records

#### SCOPUS

TITLE‐ABS (Probiotic* OR Prebiotic* OR Synbiotic*) AND TITLE‐ABS ((neonat* OR "neo nat*") OR (newborn* OR "new born*" OR "newly born*") OR (preterm OR preterm OR "pre term" OR "pre terms") OR (premature) AND (birth* OR born OR deliver*) OR (low AND (birthweight* OR "birth weight*")) OR (lbw OR vlbw OR elbow) OR infant* OR (baby OR babies)) AND NOT INDEX (medline)

#### CENTRAL

1. MeSH descriptor: [infant] explode all trees
2. MeSH descriptor: [Premature Birth] explode all trees
3. (Neonat*:ti,ab OR neo nat*:ti,ab) OR (newborn*:ti,ab OR new Born*:ti,ab OR newly born*:ti,ab) OR (preterm:ti,ab OR preterms:ti,ab OR pre term:ti,ab OR pre terms:ti,ab) OR (premature*:ti,ab AND (birth*:ti,ab OR born:ti,ab OR deliver*:ti,ab)) OR (low:ti,ab AND (birthweight*:ti,ab OR birth weight*:ti,ab)) OR (lbw:ti,ab OR vlbw:ti,ab OR elbw:ti,ab) OR infant*:ti,ab OR (baby:ti,ab OR babies:ti,ab)
4. #1 OR #2 OR #3
5. MeSH descriptor: [Animals] explode all trees
6. MeSH descriptor: [Humans] explode all trees
7. (#5 NOT (#5 AND #6))
8. MeSH descriptor: [Probiotics] explode all trees
9. MeSH descriptor: [Prebiotics] explode all trees
10. MeSH descriptor: [Synbiotics] explode all trees
11. #8 OR #9 OR #10
12. Probiotic*:ti,ab OR prebiotic*:ti,ab OR synbiotic*:ti,ab
13. #11 or #12
14. #13 AND #4 NOT #7
15. "accession number" near pubmed
16. #14 NOT #15

#### LILACS

((tw:(probiotics OR prebiotics OR synbiotics)) OR (ti:(probiotic* OR prebiotic* OR synbiotic*)) OR (ab:(probiotic* OR prebiotic* OR synbiotic*))) AND ((tw:(infant)) OR (tw:(“premature birth”)) OR (ti:((neonat* OR "neo nat*") OR (newborn* OR "new born*" OR "newly born*") OR (preterm OR preterms OR "pre term" OR "pre terms") OR (premature))) AND (ti:((born OR deliver*) OR (low AND (birthweight* OR "birth weight*")) OR (lbw OR vlbw OR elbw) OR (baby OR babies))) OR (ab:((neonat* OR "neo nat*") OR (newborn* OR "new born*" OR "newly born*") OR (preterm OR preterms OR "pre term" OR "pre terms") OR (premature))) AND (ab:((born OR deliver*) OR (low AND (birthweight* OR "birth weight*")) OR (lbw OR vlbw OR elbw) OR (baby OR babies)))) AND (instance:"regional") AND (db:("LILACS"))

### Dextrose Supplementation During Neonatal Period

#### PubMed

(((((("Glucose"[Mesh]) OR (Dextrose OR Glucose[tiab]) AND supplement*))) AND (("Infant"[Mesh] OR "Premature Birth"[Mesh]) OR (Neonat*[tiab] OR neo nat*[tiab]) OR (newborn* OR new Born*[tiab] OR newly born*[tiab]) OR (preterm[tiab] OR preterms[tiab] OR pre term[tiab] OR pre terms[tiab]) OR (premature*[tiab] AND (birth*[tiab] OR born[tiab] OR deliver*[tiab])) OR (low[tiab] AND (birthweight*[tiab] OR birth weight*[tiab])) OR (lbw[tiab] OR vlbw[tiab] OR elbw[tiab]) OR infant*[tiab] OR (baby[tiab] OR babies[tiab])))) NOT ("Animals"[Mesh] NOT ("Animals"[Mesh] AND"

#### CINHAL

(MH "Glucose") OR TI (Dextrose OR Glucose) OR AB (Dextrose OR Glucose)

AND

TI (Supplement*) OR AB (Supplement*) OR MH "Dietary Supplementation" OR MH "Dietary Supplements"

AND

(MH "Infant" OR MH "Infant, Premature" OR MH "Infant, Newborn") OR TI ((Neonat* OR neo nat*) OR (newborn* OR new Born* OR newly born*) OR (preterm OR preterms OR pre term OR pre terms) OR (premature* AND (birth* OR born OR deliver*)) OR (low AND (birthweight* OR birth weight*)) OR (lbw OR vlbw OR elbw) OR infant* OR (baby OR babies)) OR AB ((Neonat* OR neo nat*) OR (newborn* OR new Born* OR newly born*) OR (preterm OR preterms OR pre term OR pre terms) OR (premature* AND (birth* OR born OR deliver*)) OR (low AND (birthweight* OR birth weight*)) OR (lbw OR vlbw OR elbw) OR infant* OR (baby OR babies))

NOT

(MH "Animals" NOT (MH "Animals" AND MH "Humans"))

Limiter: Exclude MEDLINE records

#### SCOPUS

TITLE‐ABS (Glucose OR Dextrose) AND TITLE‐ABS (supplement*) AND TITLE‐ABS ((neonat* OR "neo nat*") OR (newborn* OR "new born*" OR "newly born*") OR (preterm OR preterm OR "pre term" OR "pre terms") OR (premature) AND (birth* OR born OR deliver*) OR (low AND (birthweight* OR "birth weight*")) OR (lbw OR vlbw OR elbow) OR infant* OR (baby OR babies)) AND NOT INDEX (medline)

#### CENTRAL

1. 1 MeSH descriptor: [infant] explode all trees
2. MeSH descriptor: [Premature Birth] explode all trees
3. (Neonat*:ti,ab OR neo nat*:ti,ab) OR (newborn*:ti,ab OR new Born*:ti,ab OR newly born*:ti,ab) OR (preterm:ti,ab OR preterms:ti,ab OR pre term:ti,ab OR pre terms:ti,ab) OR (premature*:ti,ab AND (birth*:ti,ab OR born:ti,ab OR deliver*:ti,ab)) OR (low:ti,ab AND (birthweight*:ti,ab OR birth weight*:ti,ab)) OR (lbw:ti,ab OR vlbw:ti,ab OR elbw:ti,ab) OR infant*:ti,ab OR (baby:ti,ab OR babies:ti,ab)
4. #1 OR #2 OR #3
5. MeSH descriptor: [Animals] explode all trees
6. MeSH descriptor: [Humans] explode all trees
7. (#5 NOT (#5 AND #6))
8. supplement*:ti,ab
9. MeSH descriptor: [Glucose] explode all trees
10. Dextrose:ti,ab OR Glucose:ti,ab
11. #9 OR #10
12. #11 AND #8
13. #12 AND #4 NOT #7
14. "accession number" near pubmed
15. #13 NOT #14

#### LILACS

((tw:(glucose)) OR (ti:(dextrose)) OR (ab:(dextrose)) AND (ti:(supplement*)) OR (ab:(supplement*))) AND ((tw:(infant)) OR (tw:(“premature birth”)) OR (ti:((neonat* OR "neo nat*") OR (newborn* OR "new born*" OR "newly born*") OR (preterm OR preterms OR "pre term" OR "pre terms") OR (premature))) AND (ti:((born OR deliver*) OR (low AND (birthweight* OR "birth weight*")) OR (lbw OR vlbw OR elbw) OR (baby OR babies))) OR (ab:((neonat* OR "neo nat*") OR (newborn* OR "new born*" OR "newly born*") OR (preterm OR preterms OR "pre term" OR "pre terms") OR (premature))) AND (ab:((born OR deliver*) OR (low AND (birthweight* OR "birth weight*")) OR (lbw OR vlbw OR elbw) OR (baby OR babies)))) AND (instance:"regional") AND (db:("LILACS"))
